# The relationship between media multitasking and creativity: a multi-test, multi-method analysis

**DOI:** 10.3389/fpsyg.2024.1390867

**Published:** 2024-08-14

**Authors:** Shi Chen, Han Bai, Zhicheng Zeng, Quanlei Yu, Qingbai Zhao

**Affiliations:** ^1^Hubei Health Industry Development Research Center, School of Medical Humanities, Hubei University of Chinese Medicine, Wuhan, China; ^2^Hubei Shizhen Laboratory, Wuhan, China; ^3^Key Laboratory of Adolescent Cyberpsychology and Behavior (CCNU), Ministry of Education, Wuhan, China; ^4^Key Laboratory of Human Development and Mental Health of Hubei Province, School of Psychology, Central China Normal University, Wuhan, China

**Keywords:** media multitasking, creativity, divergent thinking, convergent thinking, creative problem-solving

## Abstract

Media multitasking is widespread, yet its relationship with creativity remains unclear. This study employs a combination of measures, including the media multitasking questionnaire, alternative uses task (AUT) for divergent thinking, Chinese compound remote association task (CCRAT) for convergent thinking, and a creative problem-solving task, to examine the relationship between media multitasking and creativity. Extreme values grouping [one standard deviation above or below the mean of the media multitasking index (MMI)], median value grouping, and regression analysis were used to explore the relationship between media multitasking and creativity. The results revealed the following findings: (1) across the three analysis methods, there was no significant relationship between media multitasking and performance on the AUT task. However, within the range of one standard deviation above or below the mean of the MMI, media multitasking showed a significant positive correlation with fluency, flexibility, and total scores on the AUT task. (2) Media multitasking significantly predicted the accuracy of responses on the CCRAT task positively. (3) Media multitasking significantly predicted lower scores on the applicability of creative problem-solving tasks.

## 1 Introduction

Creativity is defined as the capacity to generate novel and valuable ideas, insights, or problem-solving strategies (Sternberg and Lubart, 1993). It is widely recognized as a crucial skill for 21st-century learners and has gained increasing attention in the field of education (Li et al., 2022). Enhancing and developing students’ creativity has long been at the center of educational goals, and is even more important in the current context of rapid AI development.

There are many factors that influence the enhancement and development of creativity in adolescents, among which the environment is an important factor (Richardson et al., 2022). The diversity of sociocultural environments is considered a fertile ground for creativity to flourish. For instance, studies have shown that long-term exposure to different cultures and lifestyles through travel and migration significantly enhances creativity (Bloom et al., 2014). In addition, the richness of the natural environment also contributes positively to the development of creativity (Palanica et al., 2019). With the rapid advancement of technology, an increasingly enriched technological environment is believed to have the potential to enhance students’ creative abilities (Henriksen et al., 2016; Yalcinalp and Avci, 2019).

The widespread use of smartphones, tablets, and laptops due to technological advancements has led to a significant increase in people’s engagement in media multitasking behaviors (Ragan et al., 2014; Carrier et al., 2015). Media multitasking refers to the simultaneous use of multiple types of media, often involving quick switches between different platforms and activities (Murphy and Creux, 2021). For instance, individuals frequently listen to music, answer phone calls, or check social media updates while using media for learning or work purposes (Shin et al., 2018). A study conducted with Chinese adolescents revealed that approximately 60.3% of them engage in media multitasking behaviors (Luo et al., 2018). As a result, media multitasking has become a topic of significant interest among educators. While some studies have found associations between media multitasking and negative learning outcomes and academic performance (le Roux and Parry, 2017; May and Elder, 2018), considering that media multitaskers need to constantly adapt to a rapidly changing flow of information in a technology-rich environment, some researchers are also increasingly interested in exploring the potential positive aspects of media multitasking, particularly its relationship with creativity.

Previous research has suggested that heavy media multitaskers (HMMs) possess individual characteristics that can facilitate creativity. Firstly, studies have shown that HMMs tend to have a higher sensation-seeking trait, actively seeking novelty and stimulation as a means to combat boredom (Jeong and Fishbein, 2007). This inclination toward adventurous strategies promotes exploratory thinking and flexible attention switching, which are considered beneficial for creativity (Friedman and Förster, 2002). Additionally, there is a significant positive correlation between sensation-seeking and various measures of creativity, such as tests of divergent thinking, picture creativity, and fantasy imagination (Okamoto and Takaki, 2010). Secondly, research suggests that HMMs have a broad attentional scope, enabling them to attend to irrelevant information more extensively (Lipsmeyer, 2009; Cain and Mitroff, 2011). This broad scope allows for exposure to a wider range of stimuli and facilitates the integration of multisensory information (Lui and Wong, 2012), which increases the likelihood of making connections between different ideas and fostering creativity (Mendelsohn, 1976). Hence, a positive relationship between media multitasking and creativity may exist.

However, HMMs may exhibit characteristics that potentially hinder creativity. Research studies have found that HMMs often have lower inhibitory control abilities (Cardoso-Leite et al., 2016; Gorman and Green, 2016). Inhibitory control refers to an individual’s capacity to regulate their attention, behavior, thoughts, and emotions, allowing them to overcome strong internal urges or external distractions (Diamond, 2013). The relationship between inhibitory control and creativity is still a subject of debate, with questions regarding whether high or low inhibitory control is more conducive to fostering creativity. However, it appears that the flexible application of inhibitory control is crucial in the creative process (Teng et al., 2018). Highly creative individuals possess the ability to quickly switch between high and low inhibitory control based on situational demands (Edl et al., 2014). The lower inhibitory control abilities observed in HMMs present challenges in maintaining focused attention and switching between different levels of inhibitory control, which can have adverse effects on creativity. Additionally, HMMs often demonstrate lower working memory capacity (Uncapher et al., 2015; Ralph and Smilek, 2017). Working memory capacity refers to the size of an individual’s working memory storage system, and reduced capacity impairs the ability of HMMs to sustain task goals and effectively manipulate information during the creative process (Chuderski and Jastrzębski, 2018).

In summary, when considering personality traits and attentional patterns, there may be a positive relationship between media multitasking and creativity. However, when considering inhibitory control and working memory capacity, there may be a negative relationship between the two. These findings suggest that the relationship between media multitasking and creativity is likely to be complex, as indicated by empirical studies exploring this association.

There appears to be a potential positive relationship between media multitasking and creative thinking, particularly in terms of divergent thinking. However, the findings from relevant studies are not conclusive. These studies typically use the media multitasking index (MMI) to represent the degree of media multitasking behavior, which reflects an individual’s tendency to handle multiple media activities at the same time. A higher MMI indicates that an individual may frequently switch between multiple apps, social media platforms, and so on; a lower MMI suggests that an individual may be more inclined to focus on one task at a time.

The results of these related studies suggest that no significant linear relationship between media multitasking and divergent thinking when analyzed using linear regression (Loh and Lim, 2020). In addition, when the heavy and light media multitasking groups were divided by 1 standard deviation above or below the mean MMI (extreme value grouping), no significant difference was found between the two groups on the divergent thinking task (Ophir et al., 2009; Gorman and Green, 2016; Loh and Lim, 2020). However, when grouped by the median value of MMI, the heavy media multitasking group had significantly higher fluency and originality scores on the alternative uses task (AUT) than the light media multitasking group (Loh and Lim, 2020). It is worth noting that the median division actually encompasses the two extreme groups under the original extreme value division. If the two extremes were removed from the high and low groups, which are divided by the median, the remaining parts should also be significantly different. Therefore, we statistically hypothesized that a positive relationship between media multitasking and divergent thinking was more likely to exist within 1 standard deviation of the MMI mean, whereas in the other ranges, no relationship existed between the two. That is, there may be a more complex nonlinear relationship between MMI and divergent thinking.

Liu et al. (2022) indeed found a staged linear relationship between MMI and divergent thinking. They used segmented regression to divide HMMs and LMMs by segmentation points. The results revealed a positive correlation between MMI and originality, flexibility, and total scores on the AUT for LMMs. However, this positive correlation disappeared for HMMs, and there was even a negative correlation between MMI and originality on the AUT. However, the observed positive correlation in their study was relatively weak, with a correlation coefficient of only 0.15 between MMI and originality on the AUT. In fact, this significant correlation may be attributed to the large sample size (*N* = 486). Additionally, if there is a positive correlation between MMI and total scores on divergent thinking for light media multitaskers, but no such correlation for HMMs, then there may be differences in the total scores of divergent thinking between the groups of HMMs and LMMs in extreme grouping. However, previous studies did not find such differences. Therefore, further exploration is needed to determine whether the relationship between media multitasking and divergent thinking is as speculated in this study or as discovered by Liu et al. (2022).

Furthermore, the relationship between media multitasking and creative thinking in terms of convergent thinking has not been established. Loh and Lim (2020) used the remote association test (RAT) to measure convergent thinking, and neither extreme value grouping nor regression analyses found significant results. However, the HMM group was found to perform significantly higher on the RAT than the LMM group when grouped by median MMI values. However, Wang (2017) also using median value grouping, failed to reproduce the above significant difference. It is worth noting that in that study, convergent thinking was the story creation task paradigm used, a paradigm that involves not only convergent thinking but also divergent thinking (Kuypers et al., 2016), so the studies reported result of no significant difference is questionable. Overall, the findings that have been made present an interesting pattern: that is, there appears to be some degree of positive association between MMI and convergent thinking under the median value grouping strategy, which is not evident in the extreme value grouping or regression analyses. There is a similarity between this pattern and the findings on the relationship between MMI and divergent thinking, so is it also the case that there is a significant positive relationship between media multitasking and convergent thinking within 1 standard deviation of the mean of MMI?

Due to the unclear relationship between media multitasking and creativity, this study utilizes the media multitasking questionnaire to calculate the MMI. By employing tasks related to both divergent and convergent thinking, the relationship between media multitasking and creativity is analyzed. It is important to note that divergent thinking and convergent thinking represent only aspects of creativity (Runco and Acar, 2012). Creative problem-solving necessitates the combined use of both divergent and convergent thinking to address ill-structured problem situations (Urban and Urban, 2023). Therefore, this study also includes a creative problem-solving task to examine its relationship with media multitasking.

## 2 Materials and methods

### 2.1 Participants

This study employed G*Power 3.1 to conduct *a priori* statistical power analysis. For the independent samples *t*-test, the statistical power index (1-*β*) was set at 0.80, with *α* set at 0.05. The analysis revealed that 128 participants were required to achieve a medium effect size of 0.5. Similarly, for the correlation analysis, aiming to detect a medium correlation of 0.3 in the current experiment, a sample of 82 participants was deemed sufficient. This study recruited 126 university students from a certain university in China as participants. After the screening, 16 individuals who did not understand the experimental instructions or did not pass the anti-random responding test in the questionnaire were excluded, resulting in a final sample of 110 eligible participants (age = 19.22 ± 1.57 years, with 97 females). All participants had normal vision or corrected-to-normal vision. Prior to the experiment, all participants signed informed consent forms and received a certain amount of compensation after the completion of the study.

### 2.2 Materials

#### 2.2.1 Divergent thinking

The AUT was employed, where participants were required to generate as many novel and practical uses as possible for three common everyday objects presented on the screen (eyeglass case, umbrella, and plastic straw) within a given time limit. Each item had a time limit of 2 min. The consensual assessment technique proposed by Amabile (1982) was used to assess the originality of participants’ responses. This method has been empirically proven to be effective (Cheng, 2018). Three trained students rated the originality of the answers on a strict 1–5 scale, with higher average scores indicating higher originality of the answers. Inter-rater reliability was assessed afterward. Fluency was calculated based on the number of valid answers, and the evaluators determined the functional dimensions of the valid answers. Flexibility was calculated based on the number of functional dimensions (e.g., “holding glasses” and “holding other things” belong to the same dimension) (Lu et al., 2021). The total scores of divergent thinking were obtained by summing the standardized scores of the three indicators from the AUT.

#### 2.2.2 Convergent thinking

The Chinese compound remote association test (CCRAT) was employed, which was selected from the aggregation thinking scale compiled by Zhang (2013) and revised by Yin (2017). In this task, participants were required to quickly generate a target Chinese character that could form a two-character word with each of the given three Chinese characters. For example, given “真, 蓝, 昨, −,” the participant needs to come up with the character “天” to form the words “天真” (pure), “蓝天” (blue sky), and “昨天” (yesterday). The target character could be placed either at the beginning or the end of the word. There were a total of 12 items in the experiment, with a time limit of 30 s for each item. The accuracy of the task was used as the measure of performance.

#### 2.2.3 Creativity problem-solving

The study employed a social event creative coping method question (Zhang, 2020). Participants were required to propose three coping strategies they considered to be the most innovative and applicable for a food safety event. Three trained students then evaluated the novelty and applicability of the answers using a strict 1–5 rating scale, with higher scores indicating greater novelty and applicability. Consistency among the raters was assessed after the evaluations were completed.

#### 2.2.4 Media multitasking

The media multitasking questionnaire proposed by Ophir et al. (2009) and revised by Loh and Kanai (2014) was used. The original 10 media tasks were slightly adjusted to align with modern lifestyles. The adjusted tasks include watching videos, listening to the audio, reading books or comics, online shopping, video or voice calls, written communication, social entertainment, learning and work-related tasks, information search or internet browsing, and online or offline gaming. Participants were required to report the total number of hours they engage in each media task per week. They then completed the media multitasking matrix questionnaire, which assessed the frequency of engaging in one primary media task while simultaneously performing another media task. The questionnaire used a 0–3 rating scale, with 3 indicating “most of the time,” 2 indicating “some of the time,” 1 indicating “occasionally,” and 0 indicating “never.” The MMI was calculated using the following formula, with a higher MMI indicating a higher level of media multitasking proficiency.


$$MMI=\overset{11}{\underset{i=1}{\sum }}\frac{m_{i}\times h_{i}}{h_{\mathrm{total}}}$$

Where m_i_ is the number of media concurrently used while using the primary medium, h_i_ is the number of hours per week spent using the primary medium, and h_total_ is the total number of hours per week using all forms of media.

### 2.3 Procedure

Participants are required to first complete the convergent thinking task, the CCRAT, and the creative problem-solving task on a computer. Following that, they will proceed to fill out the media multitasking questionnaire and provide demographic information on a mobile phone.

### 2.4 Data analysis

SPSS 26.0 was used for data analysis. Following the analysis approach by Loh and Lim (2020), three methods were employed to examine the relationship between MMI and performance on three creative tasks: (1) Extreme value grouping: participants were divided into two groups based on their MMI scores. The heavy media multitasking (HMM) group consisted of 21 individuals (*M* = 5.32, *SD* = 0.25), with MMI scores higher than the mean plus one standard deviation (MMI > 4.88). The light media multitasking (LMM) group consisted of 18 individuals (*M* = 1.93, *SD* = 0.65), with MMI scores lower than the mean minus one standard deviation (MMI < 2.61). (2) Median value grouping: participants were divided into two groups based on the median MMI score (3.80). The HMM group included 55 individuals (*M* = 4.64, *SD* = 0.60), with MMI scores higher than the median. The LMM group also had 55 individuals (*M* = 2.86, *SD* = 0.79), with MMI scores lower than the median. (3) Regression analysis: regression analysis was conducted with MMI as a continuous variable to examine its relationship with performance on creative tasks.

## 3 Results

The inter-rater reliability for scoring originality in the AUT is deemed acceptable (*α* = 0.77). Additionally, the inter-rater reliability for scoring novelty and applicability in creative problem-solving tasks is also considered acceptable (*α* = 0.78, 0.74) (Cronbach et al., 1963).

### 3.1 Alternative uses task

The extreme value method was employed to categorize participants into HMM and LMM groups. Independent samples *t*-tests were conducted to examine the differences between the two groups in terms of fluency, flexibility, originality, and total scores in the AUT. The results indicated that there were no significant differences between the HMM and LMM groups (see Table 1).

**Table 1:** Extreme value grouping.

	HMM		LMM		*t*	*p*	*Cohen’s d*
	*M*	*SD*	*M*	*SD*			
AUT fluency	16.19	5.85	17.11	3.79	−0.57	0.57	−0.18
AUT flexibility	13.67	4.64	14.94	2.73	−1.03	0.31	−0.33
AUT originality	2.73	0.62	2.96	0.36	−1.38	0.18	−0.44
AUT total scores	−0.63	3.14	0.37	1.63	−1.22	0.23	−0.39

Similarly, in the case of median value grouping, there were no significant differences observed between the HMM and LMM groups in terms of fluency, flexibility, originality, and total scores on the AUT (see Table 2).

**Table 2:** Median value grouping.

	HMM		LMM		*t*	*p*	*Cohen’s d*
	*M*	*SD*	*M*	*SD*			
AUT fluency	17.69	5.42	16.13	4.44	1.66	0.10	0.32
AUT flexibility	14.29	4.17	13.89	3.74	0.53	0.60	0.10
AUT originality	2.90	0.57	2.89	0.52	0.14	0.88	0.02
AUT total scores	0.25	2.93	−0.25	2.54	0.97	0.34	0.18

Regression analysis was conducted to examine the relationship between media multitasking and the various indicators of the AUT. The results, as shown in Table 3, indicate that media multitasking is not a significant predictor of any of the AUT indicators.

**Table 3:** Regression analyses on the relationships between media multitasking and AUT.

	*B*	*SE*	*β*	*p*
AUT fluency	0.41	0.42	0.09	0.33
AUT flexibility	0.04	0.34	0.01	0.91
AUT originality	−0.03	0.05	−0.06	0.54
AUT total scores	0.02	0.23	0.009	0.93

Based on the previous findings, this study hypothesized a positive correlation between media multitasking and performance on the AUT within one standard deviation from the mean MMI. Additionally, to minimize the possibility of chance findings, the participants were divided into groups based on the mean MMI plus/minus 0.5, 1, 1.5, and 2 standard deviations. The correlation between MMI and creativity was then analyzed, and the results are presented in Table 4.

**Table 4:** Interval correlation analysis.

	AUT fluency	AUT flexibility	AUT originality	AUT total scores
*M* ± 0.5*SD*	0.23	0.16	0.18	0.21
*M* ± 1*SD*	0.41^***^	0.27^*^	0.18	0.32^**^
*M* ± 1.5*SD*	0.13	0.07	0.02	0.10
*M* ± 2*SD*	0.08	0.001	−0.07	0.002

*p < 0.05, **p < 0.01, ***p < 0.001.

**Figure 1:**
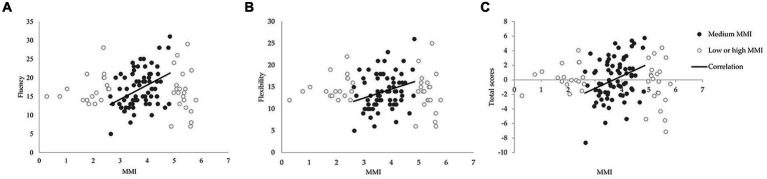
Participants were categorized into high, medium, and low groups based on the average value of media multitasking index (MMI) plus or minus one standard deviation. **(A)** Correlation of media multitasking with AUT fluency. **(B)** Correlation of media multitasking with AUT flexibility. **(C)** Correlation of media multitasking with AUT total scores.

It can be observed that only within the range of one standard deviation from the mean MMI, media-multitasking showed significant positive correlations with fluency (*r* = 0.41, *p* < 0.001), flexibility (*r* = 0.27, *p* = 0.02), and total scores (*r* = 0.32, *p* = 0.006) in the AUT. Please refer to Figure 1 for the graphical representation of these correlations.

### 3.2 Chinese compound remote associates task

In the extreme value grouping condition, the HMM group (*M* = 0.77, *SD* = 0.13) showed a significantly higher accuracy rate on CCRAT compared to the LMM group (*M* = 0.65, *SD* = 0.18), *t*(37) = 2.34, *p* = 0.03, *d* = 0.75. In the median value grouping condition, the HMM group (*M* = 0.77, *SD* = 0.11) also exhibited a significantly higher accuracy rate on CCRAT compared to the LMM group (*M* = 0.69, *SD* = 0.16), *t*(108) = 3.25, *p* = 0.002, *d* = 0.62. Regression analysis further revealed that MMI significantly and positively predicted the accuracy rate on CCRAT (*β* = 0.33, *p* = 0.001).

### 3.3 Creative problem-solving task

In the extreme value grouping condition for creative problem-solving tasks, there was no significant difference in novelty scores between the HMM and LMM groups. However, the HMM group (*M* = 2.20, *SD* = 0.54) had a significantly lower applicability score compared to the LMM group (*M* = 2.73, *SD* = 0.65), *t*(37) = −2.84, *p* = 0.007, *d* = −0.91. In the median value grouping condition, there were no significant differences in both novelty and applicability scores between the HMM and LMM groups. Regression analysis revealed that MMI could not predict novelty scores, but it significantly negatively predicted applicability scores (*β* = −0.24, *p* = 0.01).

## 4 Discussion

This study examined the relationship between media multitasking and creativity. The results indicated that there were no significant differences in the dimensions of the AUT between groups in both the extreme value grouping and median value grouping conditions. The more significant finding of this study supports the earlier speculation that within one standard deviation from the mean of the MMI, there is a significant positive correlation between media multitasking and the fluency, flexibility and total scores of AUT. In comparison to the study conducted by (Liu et al., 2022), the relationship discovered in this study is not only stronger (0.27–0.41 vs. 0.15–0.24) but also provides a reasonable explanation for the absence of group differences in the extreme value grouping and the presence of group differences in the median value grouping in previous research (Loh and Lim, 2020). Furthermore, it suggests a non-linear relationship between media multitasking and divergent thinking, demonstrated by a significant positive correlation between moderate media multitasking and divergent thinking.

The reason behind this can be explained by the aspects of sensation-seeking and attentional patterns, as mentioned earlier. Media multitasking and divergent thinking are expected to have a linear positive correlation. Firstly, individuals who engage in media multitasking tend to have higher levels of sensation seeking, which is characterized by a preference for novelty, change, and adventure and is an important trait of creative individuals (Ju, 2015). Secondly, individuals who frequently engage in media multitasking are more inclined to use a scattered attentional visual search pattern (i.e., a broader attentional scope), which may arise from the habitual parallel use of multiple media forms or rapid switching between different types of media (Lipsmeyer, 2009). Empirical research has also found that this attentional pattern can predict better performance in divergent thinking (Zmigrod et al., 2015). However, this study only found this positive relationship in the middle range, as HMMs did not exhibit higher levels of divergent thinking. This may be due to the poorer cognitive control abilities of HMMs. Previous research has indicated that HMMs categorized in extreme groups perform worse in cognitive control (Parry and le Roux, 2019), and cognitive control is closely related to divergent thinking (Teng et al., 2018). The “controlled attention theory” suggests that divergent thinking is a top-down process that requires cognitive control (Beaty et al., 2014). Empirical research has found significant positive correlations between the subcomponents of cognitive control, such as inhibitory control, and the fluency and novelty of divergent thinking (Edl et al., 2014). Working memory capacity significantly predicts the novelty of divergent thinking, and cognitive persistence partially mediates this relationship (De Dreu et al., 2012). Liu et al. (2022) also found that individuals with lower executive function (including inhibitory control) had lower levels of divergent thinking, and as their media multitasking levels increased, their levels of divergent thinking decreased. Therefore, although HMMs may have higher levels of sensation seeking and broader attentional scope, their lower cognitive control abilities may act as a counteracting factor, resulting in their relatively lower scores on AUT.

The present study investigated the relationship between media multitasking and convergent thinking. The results revealed that, overall, media multitasking levels positively predicted scores on the Chinese compound remote associate task. Consistent with the findings of Loh and Lim (2020), the study also found that, in the median split analysis, the heavy media multitasking group exhibited higher scores in convergent thinking compared to the light media multitasking group. However, in contrast to previous research, the study found that the extreme value grouping are also significantly different, and media multitasking positively predicted convergent thinking scores. The study discovered a broader positive relationship between media multitasking and convergent thinking, which could be attributed to the specific task used in the study. The Chinese compound remote associate task is a language-based measure of convergent thinking and is influenced by participants’ language proficiency and habits (Li et al., 2015). Although the study could not directly test whether higher language proficiency in HMMs led to better performance on the convergent thinking task, further analysis of the media multitasking questionnaire revealed a positive correlation between media multitasking and participants’ information search time (*r* = 0.20, *p* = 0.04). Prolonged information search may facilitate individuals’ processing of textual materials, which partially explains the overall positive correlation between media multitasking and convergent thinking. Therefore, while the differences in task paradigms (due to language and cultural background constraints, making it impossible to directly translate and use foreign-developed remote associate tests) may have contributed to the discrepancies between this study and previous research, both studies support a positive relationship between media multitasking and convergent thinking.

Finally, the study found a significant negative correlation between media multitasking and the appropriateness of creative problem-solving, indicating that individuals who engage in high levels of media multitasking tend to propose less appropriate solutions. Previous research has shown that individuals who frequently engage in media multitasking have smaller gray matter density in the anterior cingulate cortex (ACC) (Loh and Kanai, 2014). The ACC is typically associated with error or conflict detection, monitoring ongoing goal-directed behavior, providing signals when response conflicts or errors occur, and efficiently allocating attentional resources in relevant brain regions based on current task demands (Cai and Liu, 2004). This suggests that HMMs s may have lower error and conflict detection abilities, making it difficult for them to perceive inappropriate ideas when proposing viewpoints, resulting in lower appropriateness of their perspectives. Furthermore, heavy media multitaskers tend to rapidly switch between multiple media tasks, attempting to process an excessive amount of information simultaneously, potentially leading to superficial information processing or the loss of vital details. Existing research has also shown that media multitasking can impair comprehension and memory of reading materials (Armstrong and Chung, 2000; Srivastava, 2013) and negatively correlate with performance on reasoning tasks (Ma et al., 2024). Creative problem solving requires individuals to define the problem (Mumford et al., 1991), process the information in depth, and integrate the information in order to develop a more applicable perspective. Therefore, HMMs may not process the problem sufficiently and deeply enough, which makes their ideas less applicable.

It is worth noting that current research on the relationship between media multitasking and creativity is primarily based on the increasing prevalence of media multitasking behavior in the digital age. From the perspective of the creative environment, researchers attempt to explore the impact of media multitasking on creativity (Loh and Lim, 2020). However, the current empirical research is limited, and we are still in the exploratory stage of whether there is a positive relationship between the two, with most studies focusing on revealing the correlation between media multitasking and creative performance. Although existing research has not directly revealed the causal relationship between media multitasking and creativity, previous experimental studies on multitasking behavior promoting creativity can provide support for their causal relationship.

For example, Ritter and Ferguson (2017) found that participants performed better on AUT while listening to music while completing a task, but there was no difference on the convergent thinking task. Kapadia and Melwani (2021) divided participants into sequential processing groups and multitasking groups. They asked participants to reply to three emails while listening to a conference call and then complete a creative generation task and a logical reasoning task. The results showed that the multitasking group generated more novel ideas in the creative generation task, but there was no significant difference between the two groups in the logical reasoning task. This suggests that multitasking can enhance performance in subsequent creative tasks. Therefore, future research can further clarify the causal relationship between media multitasking and creativity through laboratory manipulation or longitudinal designs.

Finally, although the number of participants in this study was 110, which is relatively not large. However, this study referenced the Loh and Lim (2020) (*N* = 104) in the determination of the sample size, and the findings are relatively consistent with previous studies. Therefore, the results of this study are credible. Future research could explore the relationship between media multitasking and creativity in a larger and more diverse sample population to expand the generalizability of the current findings.

## 5 Conclusion

This study aimed to investigate the relationship between media multitasking and creativity. The following conclusions were drawn: (1) a non-linear relationship was found between media multitasking and divergent thinking. Specifically, a significant positive correlation was observed between moderate levels of media multitasking and divergent thinking, while no significant difference in divergent thinking was observed between heavy and light levels of media multitasking. (2) Media multitasking was found to be a significant predictor of convergent thinking. This implies that individuals who engage in higher levels of media multitasking tend to perform better in tasks that require convergent thinking. (3) There exists a negative relationship between media multitasking and the applicability of creative problem-solving. It was found that individuals who heavily engage in media multitasking tend to demonstrate lower levels of applicability in their creative problem-solving efforts. This suggests that the ideas generated by these individuals may be less suitable or relevant.

## Data Availability

The raw data supporting the conclusions of this article will be made available by the authors, without undue reservation.
